# Identification of biomarkers and therapeutic targets related to Sepsis-associated encephalopathy in rats by quantitative proteomics

**DOI:** 10.1186/s12864-022-09101-7

**Published:** 2023-01-04

**Authors:** Miaoxian Yang, Yu He, Yuewen Xin, Junliang Jiang, Mi Tian, Jiaying Tan, Shuixiang Deng, Ye Gong

**Affiliations:** 1 Department of Critical Care Medicine, Huashan Hospital, Fudan University, 12 Middle Wulumuqi Road, 200040 Shanghai, China; 2 Department of Neurosurgery, Huashan Hospital, Fudan University, 12 Middle Wulumuqi Road, 200040 Shanghai, China

**Keywords:** Sepsis-associated encephalopathy, Quantitative proteomics, Acute phase response, PI3K/AKT signaling, GC

## Abstract

**Background:**

Sepsis-associated encephalopathy (SAE) is a common and severe complication of sepsis. While several studies have reported the proteomic alteration in plasma, urine, heart, etc. of sepsis, few research focused on the brain tissue. This study aims at discovering the differentially abundant proteins in the brains of septic rats to identify biomarkers of SAE.

**Methods:**

The Prague-Dawley rats were randomly divided into sepsis (*n* = 6) or sham (*n* = 6) groups, and then the whole brain tissue was dissected at 24 h after surgery for further protein identification by Quantitative iTRAQ LC-MS/MS Proteomics. Ingenuity pathway analysis, Gene ontology knowledgebase, and STRING database are used to explore the biological significance of proteins with altered concentration.

**Results:**

Among the total of 3163 proteins identified in the brain tissue, 57 were increased while 38 were decreased in the sepsis group compared to the sham group. Bioinformatic analyses suggest that the differentially abundant proteins are highly related to cellular microtubule metabolism, energy production, nucleic acid metabolism, neurological disease, etc. Additionally, acute phase response signaling was possibly activated and PI3K/AKT signaling was suppressed during sepsis. An interaction network established by IPA revealed that Akt1, Gc-globulin, and ApoA1 were the core proteins. The increase of Gc-globulin and the decrease of Akt1 and ApoA1 were confirmed by Western blot.

**Conclusion:**

Based on the multifunction of these proteins in several brain diseases, we first propose that Gc-globulin, ApoA1, PI3K/AKT pathway, and acute phase response proteins (hemopexin and cluster of alpha-2-macroglobulin) could be potential candidates for the diagnosis and treatment of SAE. These results may provide new insights into the pathologic mechanism of SAE, yet further research is required to explore the functional implications and clinical applications of the differentially abundant proteins in the brains of sepsis group.

**Supplementary Information:**

The online version contains supplementary material available at 10.1186/s12864-022-09101-7.

## Background

Sepsis is a devastating organ dysfunction due to a disordered host inflammatory response to infection [1]. Sepsis remains a leading cause of health loss worldwide, with estimated 48.9 million incident cases and 11.0 million sepsis-related deaths in 2017, accounting for 19.7% of all global deaths [1]. Unfortunately, more than 70% of septic patients develop sepsis-associated encephalopathy (SAE), which is considered the most common cause of encephalopathy in intensive care unit [2]. The neurological dysfunction can last for several years in many septic survivors and severely affect their quality of life, which causes an enormous economic and social burden. SAE is a state of diffuse cerebral dysfunction without infection or structural injury in the central nervous system (CNS) [3]. Clinically, disturbance of consciousness is an early sign of SAE, with other manifestations including anxiety, depression, irritability, and a decline in cognitive function [4]. Crucial factors that might induce the development of SAE include neuroinflammation, BBB destruction, metabolism disturbance, cytokine storm, synaptic loss, and neurovascular unit dysfunction [5].

Since what determines the occurrence and persistence of SAE remains unclear, further investigations into the mechanisms underlying SAE are required. Early diagnosis and management of SAE patients are crucial to reducing disability and mortality. There is a driving need to develop biomarkers with high sensitivity and specificity to aid in early diagnosis, treatment, and ultimately improve the outcomes of SAE patients. The proteomics approach is a powerful tool to excavate such biomarkers and provide novel insight into the pathologic mechanism of SAE. Several studies have reported the proteomic changes in plasma, urine, liver, heart, etc. of both septic patients and animal models [6–8]. However, little research has focused on the proteins changing in the brain tissue of sepsis models.

**Fig. 1:**
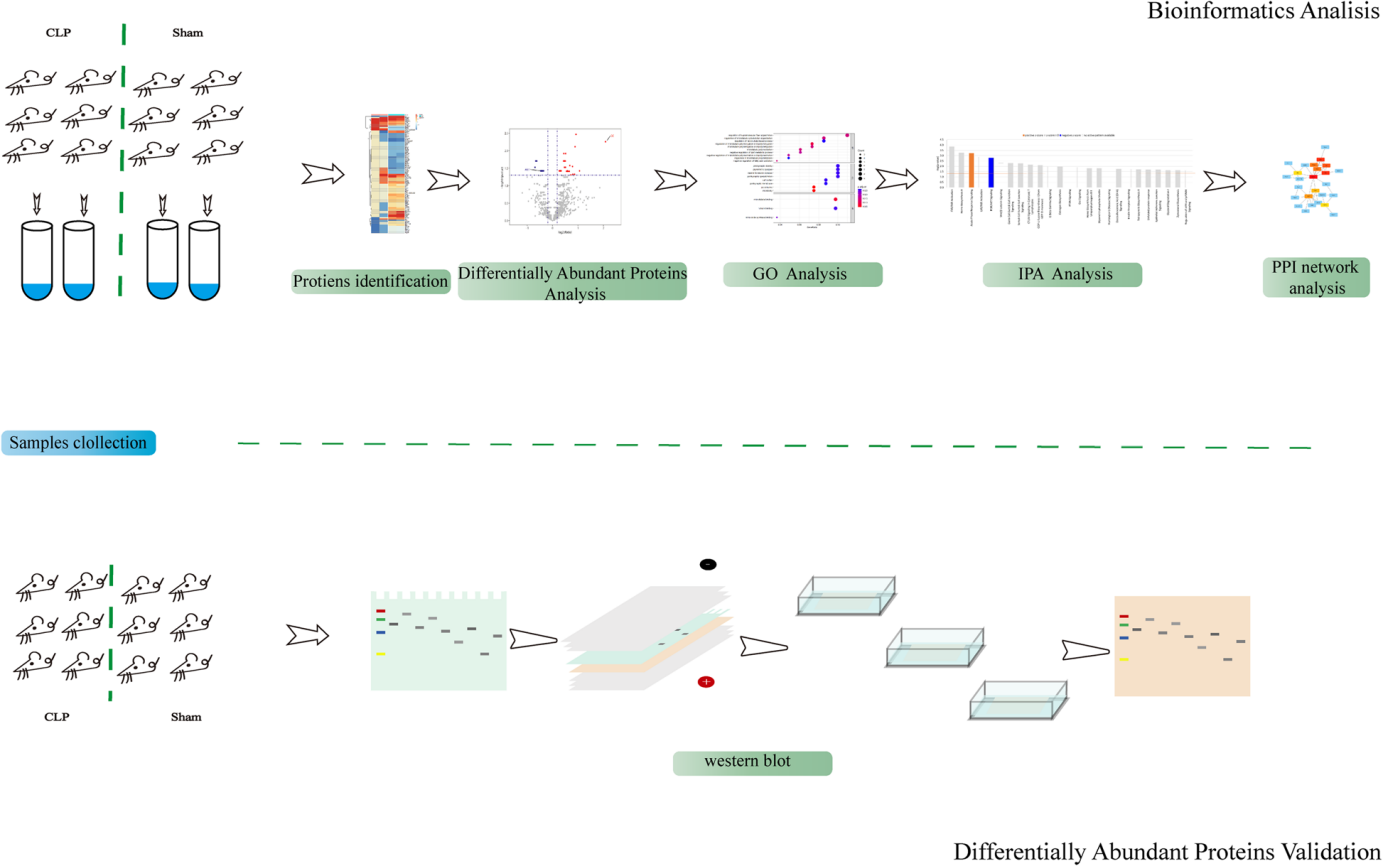
The experimental workflow. Firstly, the Prague-Dawley rats were randomly divided into sepsis (*n* = 6) or sham (*n* = 6) groups, and then the whole brain tissue was dissected at 24 h after surgery for further protein identification by Quantitative iTRAQ LC-MS/MS Proteomics. Secondly, Ingenuity pathway analysis, Gene ontology knowledgebase, and STRING database are used to explore the biological significance of the differentially abundant proteins. At last, Western blot was performed to validate the concentration of GC, Akt1, and ApoA1.

Given that the CNS is no longer suggested to be immune-privileged during sepsis [9], it is possible to hypothesize that the protein profile of the brain from the sepsis 24 h group would differ from the sham group. Through investigations into rats’ cecal ligation and puncture (CLP) models by Quantitative iTRAQ LC-MS/MS Proteomics, we aimed to discover the differentially secreted proteins in the brain of septic rats. An experimental workflow was shown in Fig. 1. This study might uncover the molecular mechanisms of SAE and guide the diagnosis, surveillance, and management of SAE patients.

## Results

### CLP model stability

**Fig. 2:**
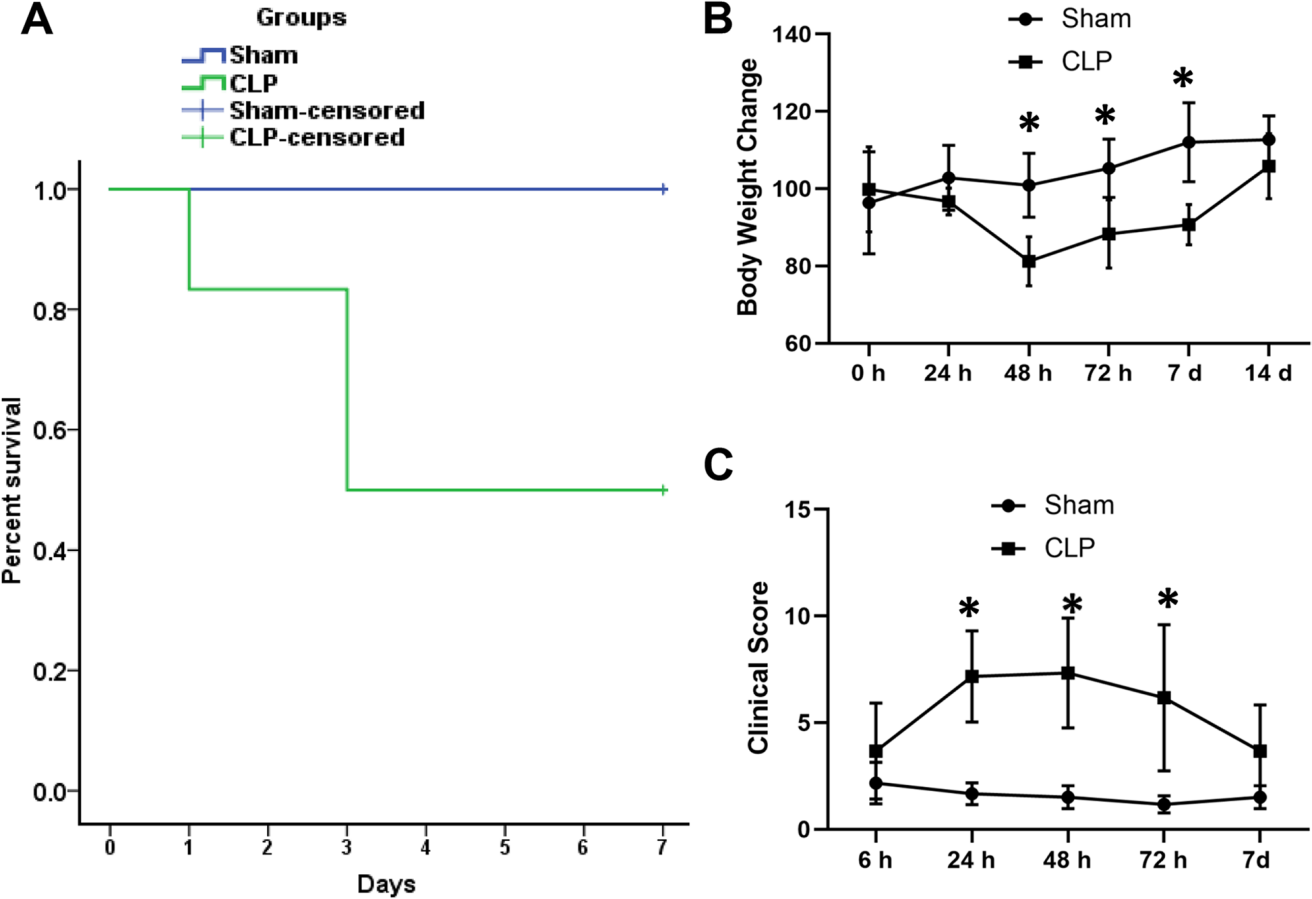
Survival, clinical score, and body weight change after CLP model. **A** Percentage of surviving animals each group 7 days after CLP, (**B**) Body weight gain in each group 14 days after CLP, *n* = 10–21/group, (**C**) Clinical score assessed each group 7 days after CLP, *p < 0.05 vs. Sham, bars represent mean ± SD.

Figure 2 depicts the survival status, clinical score, and body weight change following the CLP surgery. The CLP group had a much lower survival rate than the sham rats. The mortality rate in the CLP group is 50%. The bodyweight curve of the CLP group showed a noticeable decline at 48 h after surgery and then recovered on the 14th day. A higher clinical score was found in the CLP group, which was significantly different from the sham group from 24 to 72 h after surgery (*P* < 0.05).

### Identification and quantification of proteins in brain tissue of sepsis and sham group

**Fig. 3:**
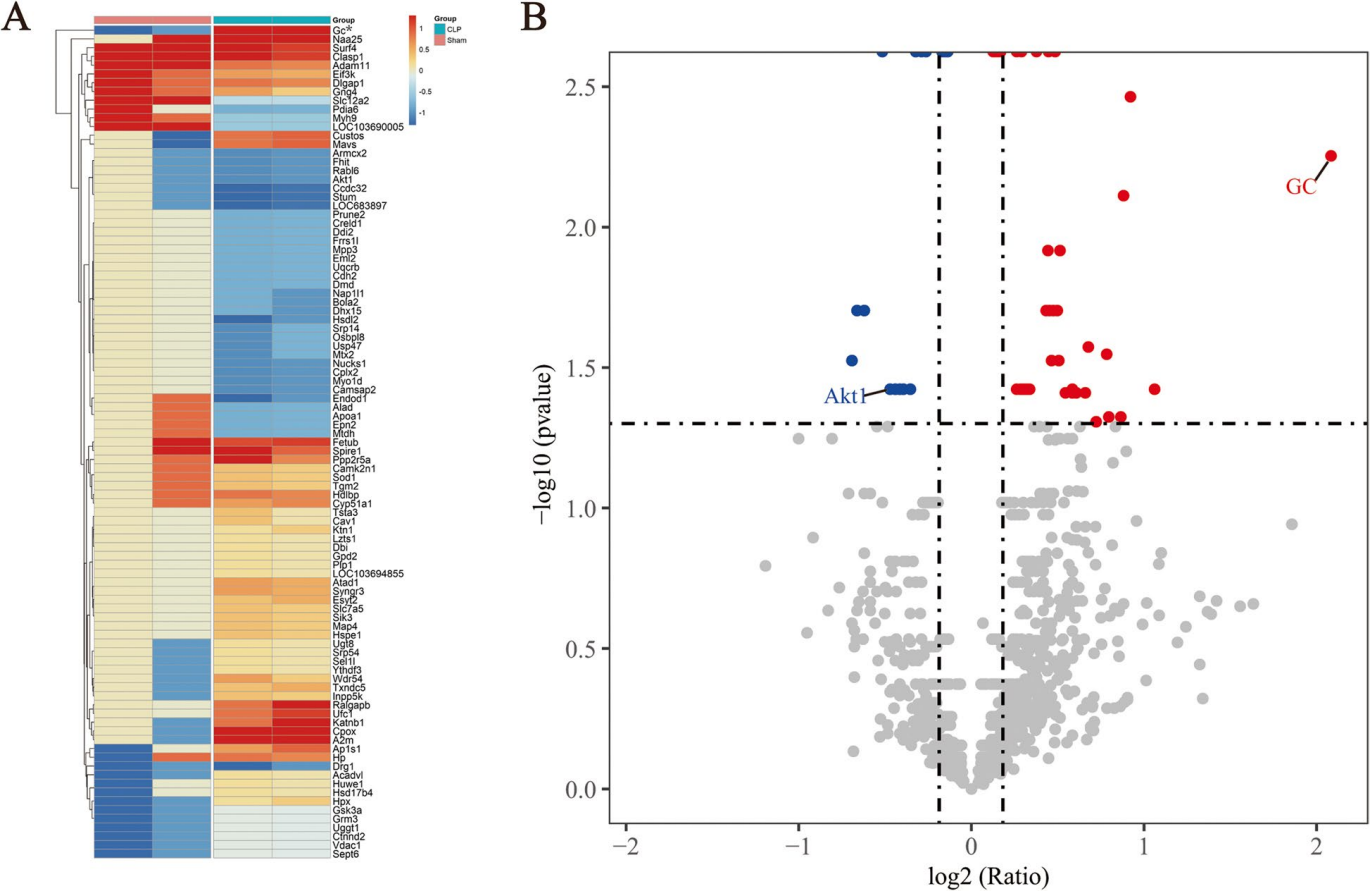
Bioinformatic analysis of proteomics data. A The heat map. Each row represents a gene and each line represents a sample dimension. The tree on the left indicates a similar settlement between genes. Color brightness is closely connected to change in expression level, with red indicating higher expression and blue indicating reduced expression. The heat map shows the 95 secreted proteins that were identified in the CLP group; of these proteins, 57 proteins increased while 38 decreased. B The volcano map. The protein ratio is represented on the x-axis, the *P*-value of the repeated test results is represented on the y-axis, and each point in the picture represents a protein. Upregulation proteins are found in the red zone, whereas downregulation proteins are found in the blue region. The volcano map reveals that a total of 3163 proteins were discovered, with 95 of the 2800 secreted proteins in the CLP group substantially differentiating from those in the control group, with 57 proteins increasing in abundance and 38 decreasing

Through the MS-based SILAC technique, we quantify the proteins released in the brain tissue to explore specific proteins that may respond to experimental sepsis at 24 h after CLP surgery. In this study, brains of 6 rats in the CLP group and 6 rats in the sham group were analyzed. 3163 proteins were identified, among which 2799 proteins were quantified (Additional file 1, 2, 3). Of them, 95 proteins were found to be differentially abundant proteins in the CLP group compared to the sham group (*p* < 0.05), with 57 proteins being increased and 38 proteins being decreased (Fig. 3A, Additional file 4). The ten most secreted proteins and the ten least secreted proteins are listed in Table 1. Gc-globulin (GC) is the most prominent of all the differentially expressed proteins, at a ratio of 4.24 times (*p* < 0.05) (Fig. 3B, Additional file 5). During sepsis, the protein expression profile in the brain is significantly altered, with the change of GC being the most noteworthy.

**Table 1:** Ten most increased proteins and ten most decreased proteins post-sepsis compared to the sham

NO	Uniprot accession	Gene symbol	Protein name	Location	Ratio	p value
1	Q68FY4_RAT	Gc	GC	Extracellular region	2.1	0.006
2	A0A0G2K9Q3_RAT	Naa25	N-alpha-acetyltransferase 25, NatB auxiliary subunit	Cytosol	1.1	0.038
3	HEM6_RAT	Cox	Oxygen-dependent coproporphyrinogen-III oxidase, mitochondrial	Mitochondrion	0.9	0.003
4	A2MG_RAT	A2m	Alpha-2-macroglobulin	Extracellular region	0.9	0.008
5	CL043_RAT	Custos	Protein CUSTOS	Nucleus	0.9	0.047
6	Q4VFZ4_RAT	Katnb1	Katanin p80 WD40 repeat-containing subunit B1	Cytoskeleton	0.8	0.047
7	MAVS_RAT	Mavs	Mitochondrial antiviral-signaling protein	Mitochondrion	0.8	0.028
8	A0A0G2K0K8_RAT	Ralgapb	Ral GTPase-activating protein subunit beta		0.7	0.049
9	B3IYD2_RAT	Ufc1	Ubiquitin-fold modifier-conjugating enzyme 1		0.7	0.027
10	B5DFI3_RAT	Ap1s1	AP complex subunit sigma	Cytosol	0.7	0.039
11	D3ZIP8_RAT	Endod1	Uncharacterized protein		-0.7	0.030
12	D4A4F9_RAT	Stum	RCG20461	Membrane	-0.7	0.020
13	M0R7R2_RAT	LOC683897	Similar to Protein C6orf203	Mitochondrion	-0.7	0.020
14	CCD32_RAT	Ccdc32	Coiled-coil domain-containing protein 32		-0.7	0.020
15	HSDL2_RAT	Hsdl2	Hydroxysteroid dehydrogenase-like protein 2	Peroxisome	-0.6	0.020
16	MYO1D_RAT	Myo1d	Unconventional myosin-Id		-0.5	0.000
17	CAMP2_RAT	Camsap2	Calmodulin-regulated spectrin-associated protein 2		-0.5	0.000
18	CPLX2_RAT	Cplx2	Complexin-2		-0.5	0.000
19	A0A0G2K7 × 3_RAT	Nucks1	Nuclear ubiquitous casein and cyclin-dependent kinase substrate 1		-0.5	0.000
20	Q5I0F0_RAT	Drg1	Developmentally-regulated GTP-binding protein 1		-0.5	0.038

The table shows proteins that are altered after sepsis and the level of alteration is expressed as log2 of the iTRAQ ratios

### Bioinformatic analysis of the proteomic data

**Fig. 4:**
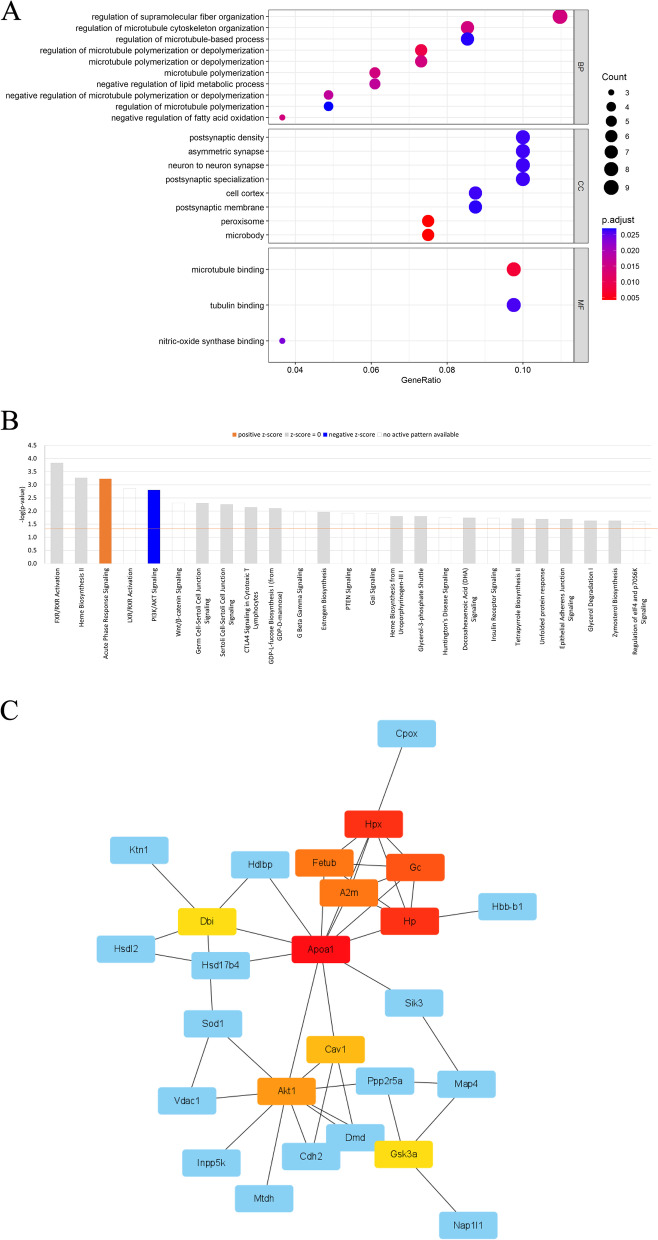
The action networks after bioinformatic analysis. **A** Dot-plot. The highest concentration of differential protein was found in five biological process items, five cellular component items, and three molecular function items, according to bioinformatic analysis. The X-axis denotes the percentage of genes that engage in the GO term, while the size of the dot indicates the count of linked genes. The *p*-value of the GO analysis is represented by the colormap. **B** The canonical pathways. Ingenuine pathway analysis of most significantly relevant pathways with the predicted activation or inhibition state. The blue bars indicate that the corresponding pathways are suppressed, and the orange bars are activated. The ratio of differentially expressed genes engaged in each pathway is represented by the orange line. **C** The PPI. Each node represents a gene for a protein, and the link between them denotes an interaction relationship. The top ten hub genes in the network are represented by colors other than blue, and the closer the color is to red, the better the score

We utilized R to perform a GO analysis on the differently abundant proteins and discovered that the top three biological processes are regulation of supramolecular fiber organization, regulation of microtubule cytoskeleton organization, and regulation of microtubule-based processes (Fig. 4A, Additional file 6). These biological processes play an important role in synaptic plasticity and cellular components, which affects neural function and the development of SAE. In addition, we further deployed the IPA to explore the canonical pathways and protein-protein interactions (Fig. 4B-C, Additional file 7). The significantly upregulated pathway is acute phase response (APR) signaling, and the significantly downregulated pathway is phosphatidylinositol-3-kinase (PI3K)/AKT signaling, indicating that the APR signaling and PI3K/AKT pathways may be crucial mechanisms in SAE. (Fig. 4B). It’s worth mentioning that the major molecule of these pathways, namely Akt1, is drastically lowered during protein quantification (Fig. 3B, Additional file 5). Additionally, the top action networks of proteins with high confidence levels and counts comprise energy production, nucleic acid metabolism, small molecule biochemistry, neurological disease, gastrointestinal disease, and hepatic system disease. Interestingly, Akt1 is also involved in these networks, namely energy production, nucleic acid metabolism, and small molecule biochemistry, while GC is related to the neurological illness network (Table 2). The STRING database was used to analyze the PPI between the differentially secreted proteins. We found that GC, Akt1, and ApoA1, linking to several proteins, are the hub proteins of the PPI networks (Fig. 4C).

**Table 2:** Major networks and proteins obtained by IPA analysis of differentially expressed proteins in sepsis group

Top diseases and functions	Molecules in network	Score focus	Molecules
Energy Production, Nucleic Acid Metabolism, Small Molecule Biochemistry	ADCY, AKT1, c-Src, CAMK2N1, caspase, CAV1, Cg, Creb, cytochrome, DBI, DHX15, FHIT, HDLBP, Hsp70, Hsp90, HSPE1, INPP5K, Lh, MAP2K1/2, MAVS, Mek, MTDH, MTX2, NAP1L1, NFkB (complex), PP2A, RALGAPB, Secretase gamma, SLC7A5, Sod, SOD1, SRP14, SRP54, USP47, VDAC1	42	20
Neurological Disease, Gastrointestinal Disease, Hepatic System Disease	A2M, Alp, AMPK, APOA1, calpain, CDH2, chymotrypsin, Collagen Alpha1, CTNND2, ERK1/2, Ferritin, GC, GPD2, HDL, HDL-cholesterol, hemoglobin, HP, HPX, JINK1/2, LDL, MAP4, NAA25, OSBPL8, p70 S6k, PDGF BB, PI3K (family), Pro-inflammatory Cytokine, SEPT6, SIK3, SLC12A2, Smad2/3, Tgf beta,TGM2, trypsin, UGGT1	31	16

### Confirmation of secreted GC and Akt1

**Fig. 5:**
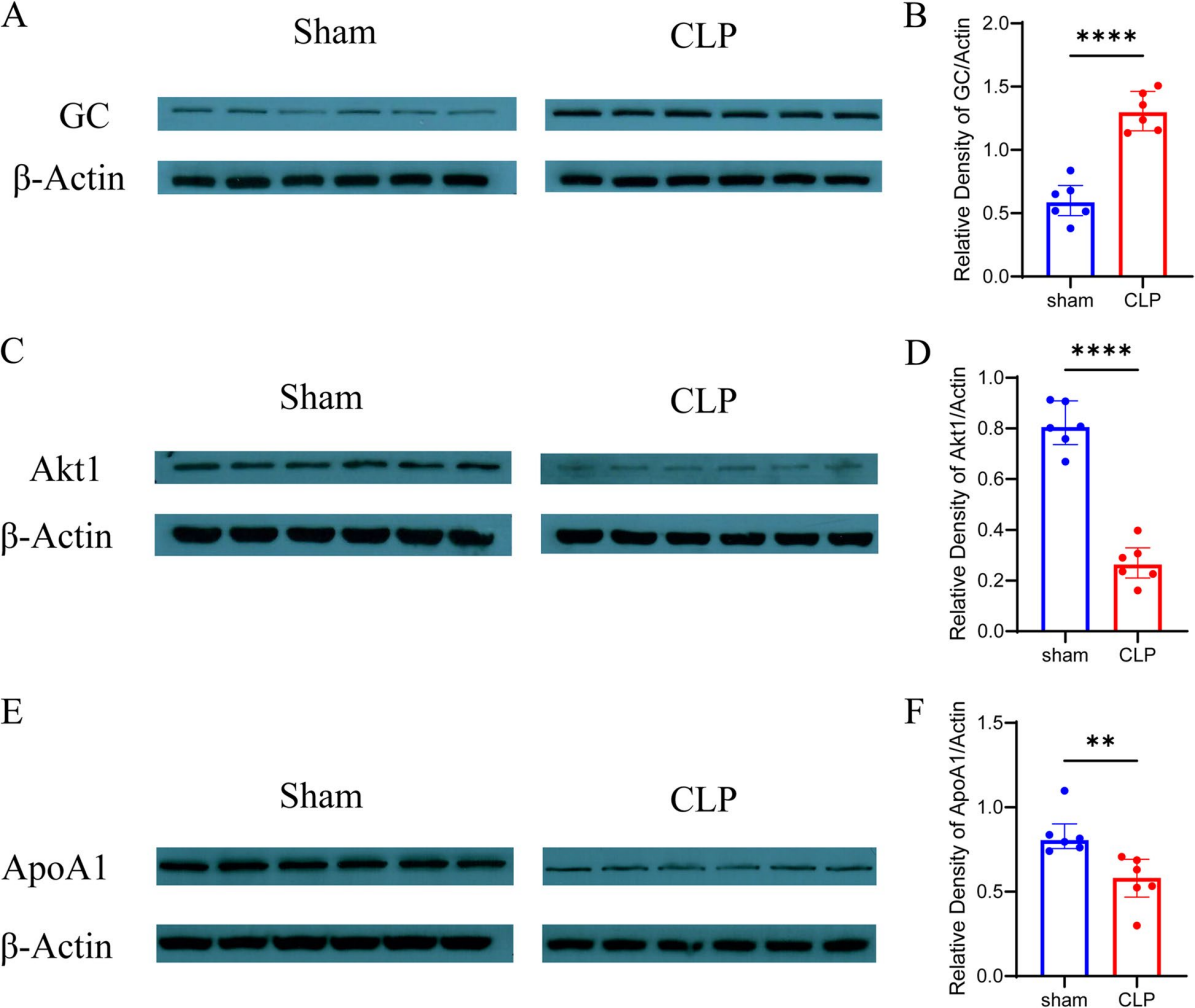
The western blot results of GC, Akt1, and ApoA1. The western blot shows a higher level of GC and a lower level of Akt1 and ApoA1 observed in the CLP group compared to the sham group (*P* < 0.05). **A**, **B** Western blot bands and quantitative analysis of GC level at 24 h in CLP and sham groups. The blots were cropped and in Additional File 8 the full-length blots are presented. **C**, **D** Western blot bands and quantitative analysis of Akt1 level at 24 h in CLP and sham groups. The blots were cropped and in Additional File 9 the full-length blots/gels are presented. **E**, **F** Western blot bands and quantitative analysis of ApoA1 level at 24 h in CLP and sham groups. The blots were cropped and the full-length blots/gels are presented in Additional File 10

The western blot validation was performed to further confirm the identification and quantification of some differentially abundant proteins. IPA and PPI analysis have revealed that GC, Akt1, and ApoA1 are the core proteins of the interaction network among the differentially abundant proteins. Additionally, GC, Akt1, and ApoA1 are highly related to the pathophysiology of SAE, including microcirculation dysfunction, neuroinflammation, BBB destruction, and metabolism disturbance [3, 5]. Therefore, we focused on the secretion profiles of GC, also known as vitamin D binding protein (DBP), Akt1, and ApoA1. The western blot findings are displayed in Fig. 5, which shows that the expression level of GC is increased, while Akt1 and ApoA1 is decreased in the CLP group compared to the sham group (*P* < 0.05).

## Discussion

CLP-induced polymicrobial infection is the most frequently used sepsis model that can closely simulate the development and characteristics of human sepsis [10]. In our study, the survival rate of CLP rats was nearly 80% at 24 h and continuously declined to 50% in the following days, which is consistent with the previous articles using the same model [11]. Additionally, obvious manifestations like piloerection, glazed eyes, chills, inertia, and the deteriorative clinical score indicated the sepsis occurring in CLP rats [12]. Given that the CNS is vulnerable to systemic inflammatory conditions, it is plausible that the development of SAE is closely associated with the early changed proteins in the brain lysate of sepsis group. Here, we used Quantitative iTRAQ LC-MS/MS Proteomics to explore the alterations in protein abundance at 24 h after CLP surgery. Among the total of 3163 proteins identified in the brain tissue, 57 were increased while 38 were decreased in the sepsis group.

IPA revealed that these differentially abundant proteins are highly related to energy production, nucleic acid metabolism, small molecule biochemistry, neurological disease, etc. These findings are consistent with previous study that glycolysis and energy metabolism may play a critical role in the course of septic encephalopathy[13]. GO analysis uncovered that the top three biological processes include supramolecular fiber organization, microtubule cytoskeleton organization, and microtubule polymerization or depolymerization. These processes are essential for neuronal morphogenesis and axonal plasticity [14]. Turbulence of the microtubule-associated proteins leads to psychiatric conditions, and neurodegenerative diseases [15]. Thus, it could conceivably be hypothesized that the neuronal damage during SAE may be associated with the destruction of cellular supramolecular fibers and microtubule cytoskeleton. Differentially abundant proteins that are involved in the regulation of supramolecular fibers and microtubules are assumed to be the promising biomarkers and therapeutic targets of SAE.

IPA also showed that most of the increased proteins were associated with the APR signaling and the decreased proteins were related to the PI3K/AKT pathway. The APR is a component of the innate immune response that responds to inflammation, injury, infections, etc. [16]. Previous studies have reported that the APR proteins was a crucial proteome change in septic liver and renal damage [17, 18]. Various APR proteins have been applied as diagnostic factors for a long time, and some APR proteins even exhibited tissue specific characteristics. It is possible, therefore, that the differently expressed APR proteins (Hemopexin, ApoA1, and Cluster of Alpha-2-macroglobulin) found in this study might be indicators of brain damage in the sepsis model. The content trend and function of these proteins are important issues for future research.

The PI3K/AKT pathway plays a crucial role in numerous bioprocesses, including apoptosis, proliferation, migration, angiogenesis, and neuroinflammation [19]. Several studies have supported that PI3K/AKT signaling is an important pathway involved in the pathological process of SAE. The effects of PI3K/AKT signaling on SAE development are diverse and depend on its activation in different cell type and various substrates [20, 21]. Research on the in vitro model of SAE showed that, through activation of the PI3K/AKT pathway, IL-1β altered the synaptic strength of GABAergic neurons and contributed to cognitive dysfunction [22]. And these effects can be interrupted by specific PI3K or AKT inhibitors [22]. A similar neuro-toxic phenomenon was obtained from an LPS-stimulated sepsis model, which showed that upregulation of PI3K/AKT signaling on BV2 microglial cells amplified the inflammatory cascade [23]. However, some studies have suggested that activation of PI3K/AKT signaling could mediate neuronal survival during sepsis. Various biomolecules and pharmaceuticals, such as Chromogranin A-derived peptide CGA47-66, Ghrelin, Pristimerin, and Dexmedetomidine, could attenuate blood-brain barrier (BBB) destruction [24], brain edema [25], neuroinflammation [25], synaptic injury [26], neuronal apoptosis [27, 28]. Thus, PI3K/AKT signaling could represent potential diagnosis markers, therapeutic targets, and prognosis indicators in neural dysfunction during sepsis.

Of the 57 increased proteins, we focused on the GC (also called DBP), which is a multifunctional protein and a potential biomarker in sepsis assessment. Interestingly, while several studies showed a decreased level of GC in the plasma of sepsis patients and rats [29–31], this might be the first study to find that GC was significantly increased in the brain of the sepsis rat models. The western blot was used to confirm the higher expression of GC in the brain of the sepsis group. The potential source of the elevated GC might be attributed to local release in brain tissue rather than periphery organs. Research on the brains of Alzheimer’s disease (AD) patients and transgenic AD model mice reported that GC was increased in the cerebrospinal fluid and brain tissue. It can reduce the aggregation of amyloid beta and rescue the memory deficits of AD mice [32]. The lower plasma concentration of GC is correlated with a higher risk of multiple organ dysfunction [33], septic shock [34], and in-hospital mortality [35]. However, until now, no studies have explored the role of GC in the pathophysiology of SAE.

The pathophysiology of SAE is not clear, likely mechanisms include microcirculation dysfunction, neuroinflammation, BBB destruction, metabolism disturbance, and alterations in neuronal synapses [3, 5]. GC is associated with the pathophysiologic processes mentioned above, which makes it possible to be a diagnosis biomarker, prognostic factor, and therapeutic target of SAE. GC belongs to the albuminoid superfamily. In addition to its well-recognized function of transporting vitamin D [36], numerous papers have revealed that GC also played important role in (1) scavenging actin, (2) binding endotoxins, (3) enhancing the chemotaxis of neutrophils, and (4) regulating the activity of macrophages and T cells [37]. Through binding fatty acids and actin monomers, GC prevents the polymeric actin fibrils from blocking vessels [38]. Patients with severe neurodegeneration were shown to increase the intrathecal synthesis of GC that is able to clear the actin leaked from the damaged cells and prevent intravascular coagulation [39]. Thus, it might protect against microcirculation dysfunction and reduce the cerebral microinfarcts that occur in sepsis patients [40]. Elevated blood endotoxin in the circumventricular organs, choroid plexus, and leptomeninges during sepsis can activate microglia and then damage neurons by releasing inflammatory factors and phagocytosis of synapses [5, 41]. The ability of GC to bind endotoxin was confirmed by experiments of radioimmunoprecipitation and isoelectric focusing [42].

The effects of GC on inflammation regulation are intricate. GC enhances the chemotaxis of neutrophils partly through the complement factor 5-mediated pathway and increases the activation of microglia by its selective deglycosylated variant, Gc-macrophage activating factor (Gc-MAF) [43, 44]. Evident microglia activation and cerebral recruitment of neutrophils were ubiquitous in SAE, which evoked an inflammatory cascade and caused further brain edema and neuron death [45, 46]. However, in vitro experiments on RAW 264.7 macrophages suggested that Gc-MAF can also induce macrophage apoptosis when the activated macrophages are excessive during inflammation [47]. Additionally, a commercially available formula of Gc-MAF, which has already been used in the immunotherapy of advanced cancers, was reported to reverse the toxic effects of oxaliplatin on human neurons (SH-SY5Y cells) but did not alter the anticancer effect on human microglial cells (C13NJ) [48]. As for T cells, the influence of GC on T cell response is varied depending on the concentration and phenotypes of GC, 25(OH)-vitamin D3 concentration, vitamin D receptor subtypes, etc. [37]. Cerebrally infiltrated Treg and Th2 cells were showed to mitigate neuroinflammation and attenuate mental impairment in the cecal slurry-induced septic mouse model[49]. Further research is needed to investigate the crosstalk between T cells and GC and its effects on the pathologic process of SAE. Increased permeability of the BBB occurs in sepsis, which enables the peripheric GC to infiltrate into the brain and exert clearance of actin and endotoxins [39]. Pre-clinical toxicology experiments of human Gc globulin have suggested that its safety profile was consistent with that required for use in humans [50]. Therefore, we suggest that GC is a potential biomarker to assess brain damage during sepsis and a promising therapeutic target to eliminate endotoxins and regulate immunocompetent cells during SAE.

IPA was used to establish an interaction network among the differentially abundant proteins. It revealed that Akt1, GC, and ApoA1 are the core proteins, among which ApoA1 is the key regulator linking Akt1 with GC. To our best knowledge, this might be the first article to report that in the brain of SAE rats, ApoA1 was decreased. Decreased plasma concentrations of ApoA1 were detected in severe septic patients and non-survivors of septic dogs [51, 52]. ApoA1 is a major component of high-density lipoproteins with pleiotropic functions including anti-inflammation, antioxidant, anti-apoptosis, etc.[53]. While most studies investigated the expression and functions of ApoA1 in cardiovascular diseases and lipid metabolism, the impacts of ApoA1 in SAE remain to be explored. Several studies have indicated that, in AD, intravenous administration of ApoA1 can reduce Aβ levels and alleviate neuroinflammation [54]. Additionally, research on neuroblastoma-associated neuronal injury models has shown that ApoA1 is initially diminished and then sharply increased in response to neuronal injury. It acts as a self-protecting mechanism that promotes wound healing after neuronal injury [55]. Therefore, it seems that ApoA1 is a potential therapeutic target and prognosis indicator.

Due to practical constraints, this paper only provided proteomic changes of the brain tissue at 24 h after sepsis in rats. Additionally, the whole brains of septic rats were dissected for further analysis, which neglected the possibly diverse changes in different brain regions. In future investigations, it might be important to uncover the proteomic changes in the different disease phases and different brain areas of SAE. Besides, further preclinical and clinical studies are required to explore the function and clinical application of these differentially abundant proteins.

## Conclusion

In this proteomic analysis of brain tissue in the septic rat, we discovered that 57 proteins were increased while 38 were decreased in the sepsis group. The western blot was used to confirm the changed concentration of GC, Akt1, and ApoA1. The differentially abundant proteins are involved in the processes of cellular microtubule metabolism, energy production, nucleic acid metabolism, neurological disease. These results may provide new insights into the pathophysiologic mechanism of SAE, yet further research is needed to explore the functional implications and clinical applications.

## Method

### Animals

The experimental design and animal methodologies for this research were approved by the Animal Experimental Committee of Huashan Hospital Fudan University’s Animal Care Guidelines. In this experiment, 250–300 g Sprague-Dawley rats were purchased from Shanghai SLAC Laboratory Animal. The mice were kept in temperature and humidity-controlled conditions and had free access to water and food. The light in the room is limited to a 12-hour light/dark cycle. Mice were randomly assigned to each group, and random numbers and a unique code associated with a single animal were generated using SPSS version 18.0 software.

### Experimental design

There are two separate experiments and the summary of experimental groups are shown as following. Proteomic analysis, western blot validation and clinical scores evaluation were assessed in a blinded manner. The investigators were blinded for the surgical procedures and treatment. S.X.D. performed surgeries, and M.X.Y. and Y.H. did all other measurements.

#### Experiment 1

To quantify the protein expression in the brain at 24 h after septic encephalopathy. The rats were divided into CLP group and Sham group at random (6/group) for proteomic analysis after identify the CLP model stability.

#### Experiment 2

To validate the proteomic results, the rats were divided into CLP group and Sham group at random (6/group). After identify the CLP model stability, we used immunoblotting to test GC and Akt1 expression in both groups of mice.

### CLP Models

In this experiment, 250–300 g Sprague-Dawley rats were employed. Brains of 6 rats were harvested at 24 h after CLP. Similar surgical procedures were performed on 6 sham animals without generating CLP. Sodium pentobarbital (40 mg/kg intraperitoneal injection) was used to anesthetize the rats. Make a 3-4 cm longitudinal skin midline incision to get access to the peritoneal cavity. At the middle of the ileocecal valve and distal cecum, 6.0 silk thread (6 − 0 PROLINE, 8680 g; Ethicon) was firmly ligated. Perforate the cecum with a single through-and-through puncture in a mesenteric-to-antimesenteric orientation, halfway between the ligation and the tip of the cecum. Extrude a small amount (droplet) of feces from both the mesenteric and antimesenteric penetration holes after withdrawing the needle to confirm patency. Skip these steps from constricting the cecum for sham animals. Move the cecum into the abdominal cavity and suture the skin. The animal was placed in an incubator with free access to food and water after the incision was sutured. Subcutaneously administer prewarmed normal saline (37 °C; 5 ml per 100 g body weight) to resuscitate the animals. The rats were sedated by sodium pentobarbital at 24 h after the operation. The brains were taken after anesthesia and sectioned coronally at the level of the optic chiasm (2 mm posterior to the chiasm), resulting in one section. Whole brains were dissected quickly, washed in ice-cold PBS, snap-frozen in liquid nitrogen, and retained at -80 °C until used.

### Proteomic analysis

#### Protein extraction

After the brains of 6 rats per group were harvested at 24 h after CLP or sham, three brains in each group were mixed into one tube for quantitative analysis of protein, and finally the relative protein concentration of each tube was obtained. The material was resuspended in a lysis solution that was roughly eight times its original volume (4% SDS, 100 mM Hepes, pH = 7.6, phosphatase inhibitor cocktail, and PMSF). On the ice, the homogenate was sonicated for 10 min. The supernatant was stored at -80 °C after centrifugation at 25,000 g for 30 min at 4 °C. A BCA kit was used to determine the total protein content.

#### In-solution digestion/labeling

The extracted proteins were divided into groups and blended in equal amounts before being precipitated overnight with acetone. Protein quantification was conducted using the BCA Kit after resuspending the proteins in the TEAB buffer. The proteins were reduced by 5 mmol/L DTT for 30 min at 56 °C and alkylated by 10-mmol/L IAA for 30 min at room temperature. The samples were then diluted with ammonium bicarbonate (50 mmol/L) until the urea content was less than 1 M. For 12 h, trypsin was administered to the samples at a 1:50 mass ratio (enzyme: protein). The peptide samples were incubated at room temperature for 120 min with iTRAQ-8plex labeling reagents (AB Sciex). With the addition of water, the reaction was halted, followed by concentration using SpeedVac and desalting. The pure peptides were collected and kept at -80 °C until they were needed.

The peptides were fractionated using a C18 column (Waters BEHC18 2.1 50 mm, 1.7 m) in a water UPLC. Peptides were eluted using a linear gradient of 5–35% solvent B (acetonitrile) over 10 min at a flow rate of 600 L/min; solvent A is 20 mM ammonium formate with pH adjusted to 10. At 214 nm, the absorbance was measured. 12 fractions were collected and lyophilized.

#### LC-MS/MS analysis

Each fraction was separated using a reverse-phase analytical column (Eksigent, C18, 3 m, 150 mm, 75 m) and nano-HPLC (Eksigent Technologies). Peptides were then eluted using the gradient conditions below, using phase B (98% CAN with 0.1% formic acid) from 5 to 45% phase B (5–78 min) with a total flow rate of 300 nL/min. A 2.5 kV electrospray voltage was applied to the mass spectrometer’s intake. The information-dependent data collection mode on the 5600 mass spectrometer was used to switch between MS and MS/MS acquisition automatically. The mass range of 350–1250 m/z was covered by MS spectra. With a dynamic exclusion time of 30 s, the twenty most powerful precursors were chosen for fragmentation every cycle.

#### Data processing

Database Searching Mascot was used to evaluate all MS/MS samples (Matrix Science, London, UK; version 2.3.0). Mascot was programmed to search the UniProt Knowledgebase (https://www.uniprot.org/taxonomy/9606), Taxonomy - Homo sapiens (205,050 sequences, 20,399 reviewed and 184,651 unreviewed), assuming trypsin digestion. Mascot was searched with a 0.1 Da fragment ion mass tolerance and a 20.0 ppm parent ion tolerance. Mascot defined cysteine carbamidomethyl and lysine iTRAQ8plex, as well as the N-terminus, as permanent alterations. Methionine oxidation and the iTRAQ8plex of tyrosine were listed as changeable alterations in Mascot.

Criteria For Protein Identification To validate MS/MS-based peptide and protein identifications, Scaffold (version Scaffold 4.4.5, Proteome Software Inc., Portland, OR) was employed. The Scaffold Local FDR method approved peptide identifications with an FDR of less than 1.0%. Protein identification was allowed if the FDR was less than 1.0% and at least one peptide was found. The Protein Prophet program gave probability to proteins (Nesvizhskii et al. 2003). To meet the concept, proteins with identical peptides that could not be distinguished by MS/MS analysis alone were grouped.

Scaffold Q+ (Proteome Software Inc., Portland, version Scaffold 4.4.5) was used to quantify iTRAQ peptides and protein identifications. The experiment’s acquired intensities were globally adjusted across all acquisition runs. Within each acquisition run, individual quantitative samples were standardized. Within the designated protein, the intensities for identification of each peptide were standardized. A 1:1-fold change was achieved by normalizing the reference channels. To multiplicatively normalize data, all normalization computations were conducted using medians.

#### Bioinformatics

We utilized all of the rat proteins as the foundation for determining enrichment values for gene ontology (GO) analysis, which was done using a self-written R script. The significance of the enrichment values, which were then depicted as a heat map, was tested using a two-tailed Fisher’s exact test and an FDR control. The Kyoto Encyclopedia of Genes and Genomes (KEGG) pathway database was also used to undertake pathway analysis.

Ingenuity pathway analysis (IPA; Ingenuity R Systems, www.Ingenuity.com/) was used to examine canonical pathways and linkages within the uploaded data to better understand the biological implications of differentially expressed plasma proteins. The importance of each canonical route was determined using the right-tailed Fisher’s exact test, with P values less than 0.05 deemed statistically significant. With differently expressed proteins and a Z score, disease and functional protein networks, as well as upstream regulator analyses with differentially abundant proteins, were presented. Significant activation or inhibition was defined as a Z score of 2 or -2, respectively.

#### Protein-protein interaction

Protein-protein interaction (PPI) is obtained by analyzing all differentially abundant proteins in the database, STRING (https://cn.string-db.org/) with default parameters, and further processing in the software, Cytoscape (Version: 3.9.0). The STRING is a database aimed to analysis all known and predicted associations between proteins. Cytoscape is an open-source software platform for visualizing complex networks and integrating them with any type of attribute data. The plug-in cytoscape, cytohubba, assigns a value to each gene using a topological network algorithm (NNC) with default parameters, ranking it to discover its key gene (hub gene).

### Confirmation of protein expression by western blot validation

Western blot was used to validate the LC-MS/MS results of chosen proteins in brain homogenates from sham and CLP rats. Brain homogenates were separated on 12.5% polyacrylamide gels (Bio-Rad, Hercules, CA, USA) and then transferred to a PVDF membrane in equivalent protein levels (from 50 to 80 g depending on each individual protein) (Millipore). Membranes were blocked for 1 h in TBS-T buffer containing 5% skim milk, then incubated overnight at 4 °C with the following antibodies: rabbit GC (1:3000, Abcam, USA), rabbit Akt1 (1:1000, Abcam, USA), rabbit ApoA1 (1:1000, Abcam, USA), primary β-actin antibody (1:3000, Abcam, USA), and secondary antibody (goat anti-rabbit IgG conjugated to horseradish peroxidase (1:5000, Abcam, USA). Chemiluminescence of the substrate luminal reagent was used to visualize the particular reaction (GE Healthcare, UK). After that, the blot photographs were scanned. ImageJ software (Image J 1.53t, NIH, USA) was used to measure the band intensity of each sample. To normalize the protein expression, the intensity of the target protein is divided by that of the loading control. And then relative target protein expression was compared to assess changes in target protein expression across samples. Each group consisted of six rats.

### Clinical scores

The rats’ clinical scores for sepsis were measured using a modified SHIRPA technique (SmithKline Beecham, Harwell, Imperial College, Royal London Hospital, Phenotype Assessment). Behavioral features (activity, posture, and responsiveness to stimuli) were examined, as well as fur properties, respiratory rhythm, body temperature, fecal attributes, and body weight. The rats were given a potential score ranging from 1 to 12 for each characteristic.

### Statistical analysis

All statistical analyses were performed using SPSS for Windows, Version 18.0. The mean and standard deviation of normally distributed variables are shown (SD). To compare protein abundance between groups, an unpaired Student’s t-test was used. Fold change was calculated using the protein abundance of the control group as a reference; changes of 1.2 or more and P values of 0.05 were considered significant.

## Supplementary Information

**Additional file 1.** Raw data. 

**Additional file 2.** Peptide report.

**Additional file 3.** Protein report.

**Additional file 4.** Heatmap.

**Additional file 5.** Volcanomap.

**Additional file 6.** GO analysis

**Additional file 7.** Canonical pathways. 

**Additional file 8.** Full-length blots of GC (also named as DBP).

**Additional file 9.** Full-length blots of Akt1.

**Additional file 10. **Full-length blots of ApoA1.

## Data Availability

The datasets of mass spectrometry proteomics generated during the current study are available in the PRIDE repository, with the dataset accession number PXD035112. This data is inaccessible until the publication of the corresponding manuscript, so please use the reviewer account to view this data: Username: reviewer_pxd035112@ebi.ac.uk. Password: k114i0qH.

## Abbreviations

SAE: sepsis-associated encephalopathy
CLP: cecal ligation and puncture
CNS: central nervous system
GO analysis: Gene ontology analysis
GC: Gc-globulin
APR: acute phase response
BBB: blood-brain barrier
DBP: vitamin D binding protein
AD: Alzheimer's disease
GC-MAF: GC-macrophage activating factor
IPA: ingenuity pathway analysis
PPI: protein-protein interaction
