# Supplementary figures and images for: Identification of biomarkers and therapeutic targets related to Sepsis-associated encephalopathy in rats by quantitative proteomics
Source: BMC Genomics. 2023 Jan 4;24:4. doi: 10.1186/s12864-022-09101-7 (PMC9814352; doi:10.1186/s12864-022-09101-7)

Sham

CLP

GC

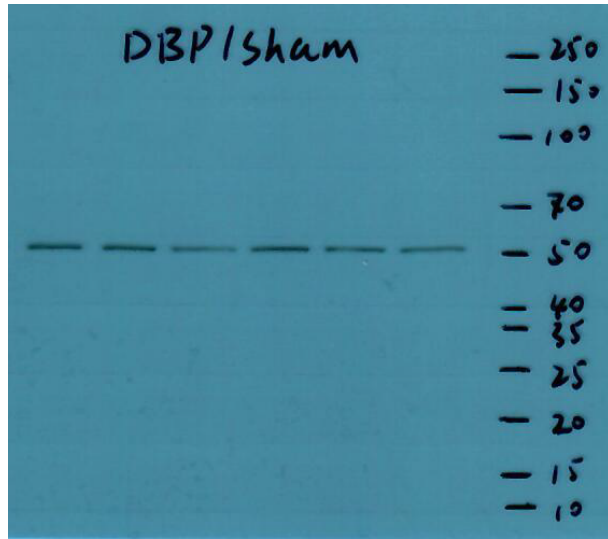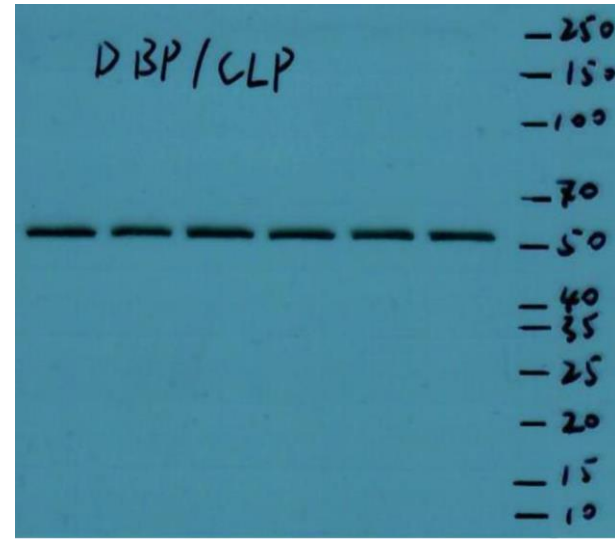

$\beta$ -Actin

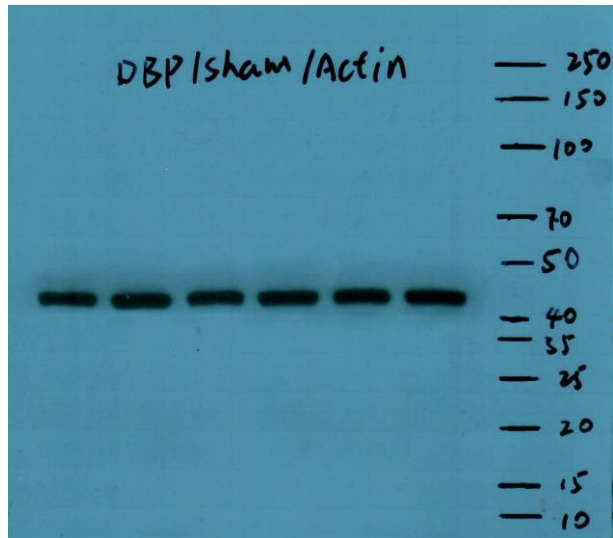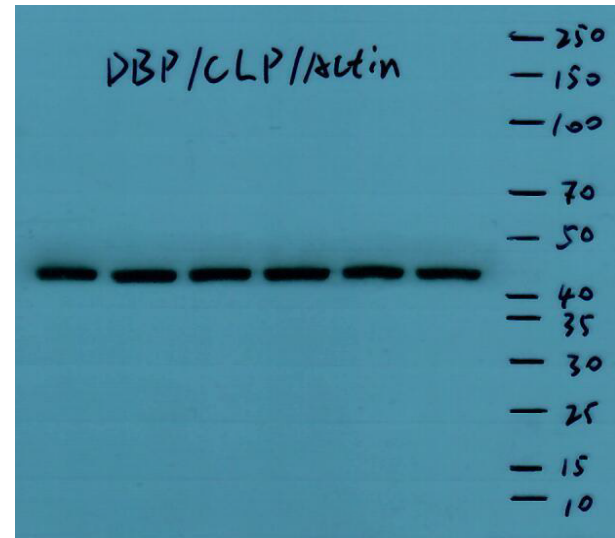

Supplement: Supplementary file 8 — Additional file 8. Full-length blots of GC (also named as DBP). [file 12864_2022_9101_MOESM8_ESM.pdf]

Sham

CLP

Akt1

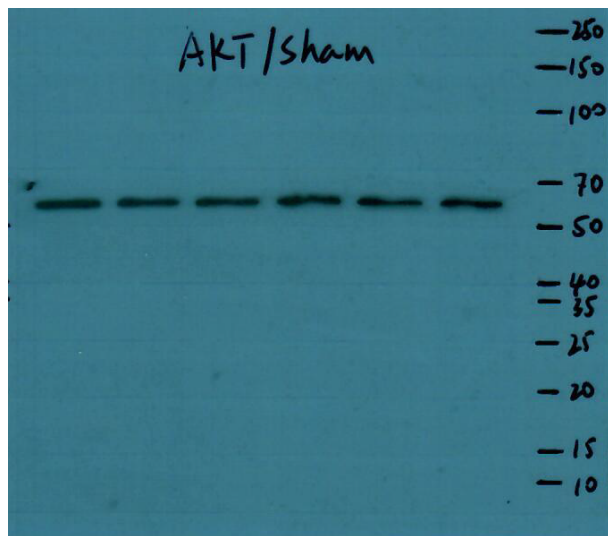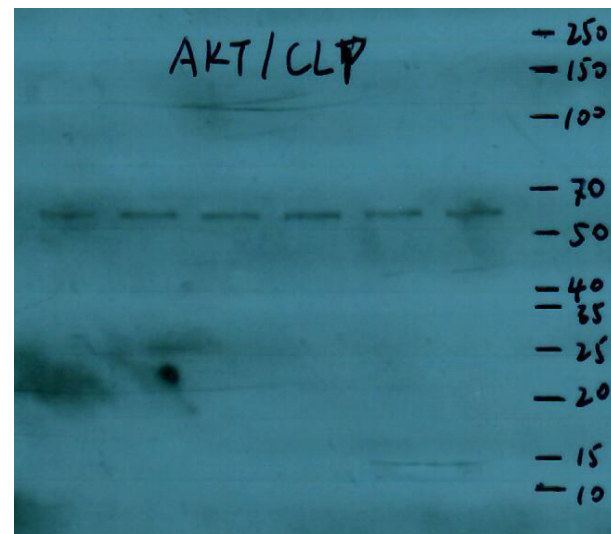

$\beta$ -Actin

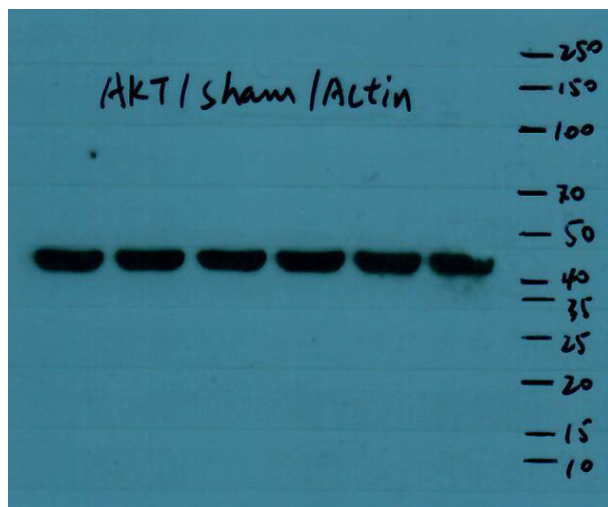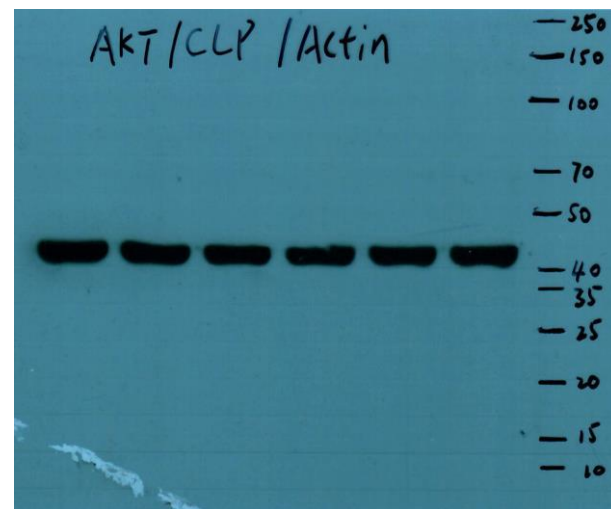

Supplement: Supplementary file 9 — Additional file 9. Full-length blots of Akt1. [file 12864_2022_9101_MOESM9_ESM.pdf]

Sham

CLP

ApoA1

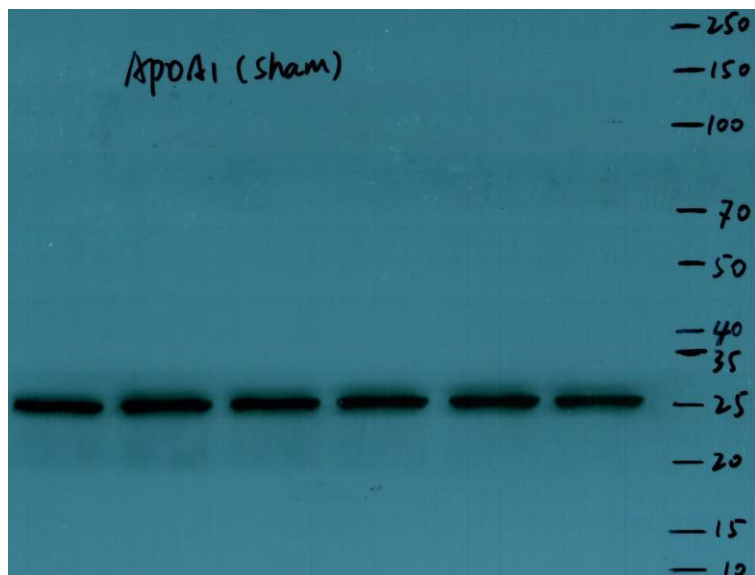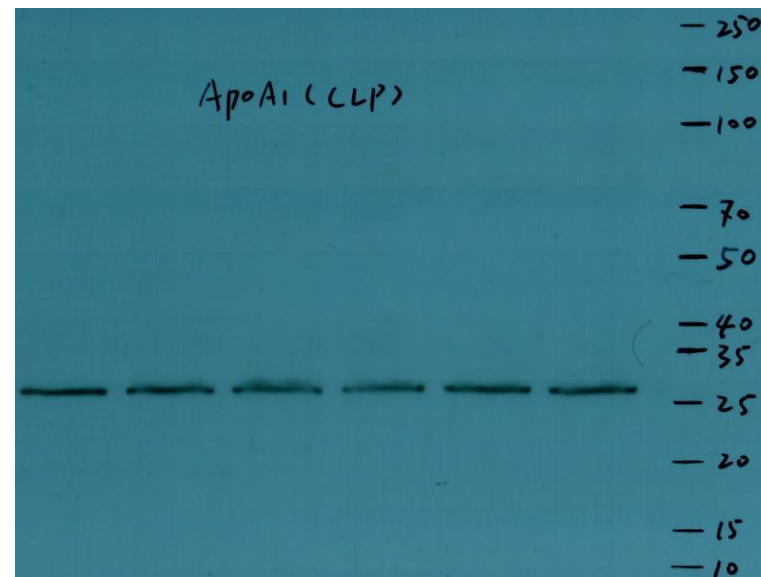

$\beta$ -Actin

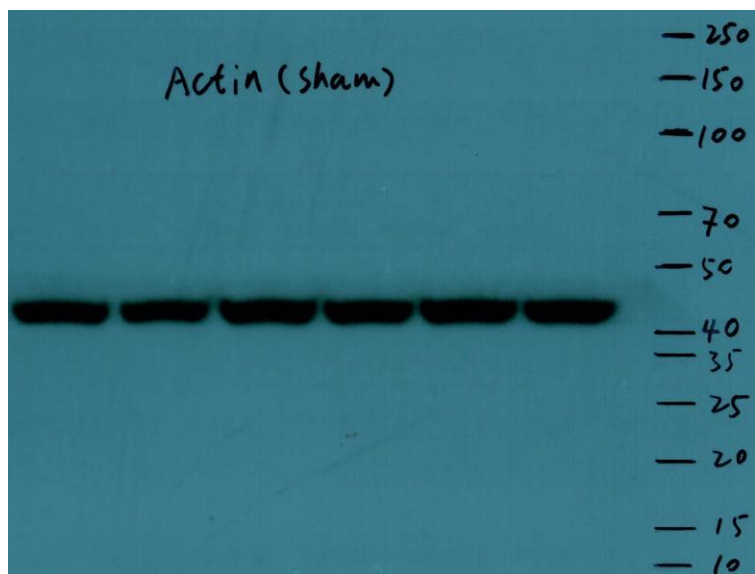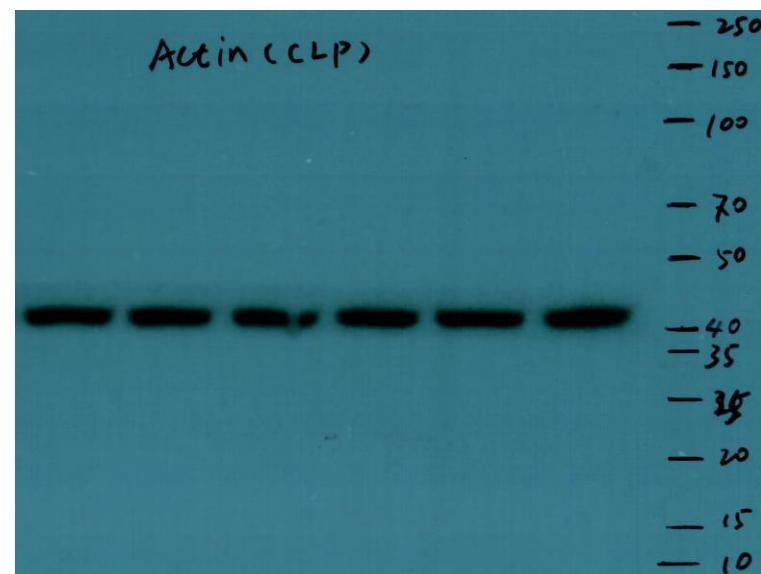

Supplement: Supplementary file 10 — Additional file 10. Full-length blots of ApoA1. [file 12864_2022_9101_MOESM10_ESM.pdf]
